# Towards the complexity of laugh communication in great apes: exact facial replications in laugh faces of orangutans and chimpanzees

**DOI:** 10.1038/s41598-026-43992-w

**Published:** 2026-03-14

**Authors:** Diane A. Austry, Kim Bard, Violet Gibson, Hélène Chotard, Alice Judge, Cristina Costantini, Guillaume Dezecache, Marina Davila-Ross

**Affiliations:** 1 School of Psychology, Sports and Health Sciences, University of Portsmouth, Portsmouth, UK; 2 Department of Psychology, Durham University, Durham, UK; 3 Department of Behavioral Sciences, University of Michigan-Dearborn, Dearborn, Michigan USA; 4 Southampton Solent University, Southampton, UK; 5 Royal (Dick) School of Veterinary Studies, University of Edinburgh, Edinburgh, UK; 6 Department of Psychology, Faculty of Life and Health Sciences, Oxford Brookes University, Oxford, UK; 7 Université Clermont Auvergne, LAPSCO, CNRS, Clermont-Ferrand, France

**Keywords:** Facial communication, Laugh faces, Rapid facial mimicry, Facial replication, Social play, Orangutans, Chimpanzees, Biological anthropology, Human behaviour, Animal behaviour, Behavioural ecology

## Abstract

Exact facial replications, where individuals match the same facial variant type (e.g., Duchenne smiles) of others, play a central role in everyday social interactions of humans. Such replications help to improve an understanding of others by allowing individuals to be emotionally more in tune with them and/or to predict their behaviours. In this study, we tested for exact facial replications in orangutans and chimpanzees – the great apes phylogenetically farthest from and closest to humans, respectively. We tested in 96 subjects (from altogether eight social groups) whether such facial replications occurred within three seconds for laugh faces, homologues of human smiles/laugh faces. We focused on two variant types (laugh faces with and without the upper teeth exposed), using a two-step method. Our study revealed that both the orangutans and chimpanzees showed exact facial replications for laugh faces where the upper teeth were not exposed, i.e., the variant type that was least associated with rough/risky play. Such variant preference for exact replications might help being emotionally more in tune with others and/or predict their behavioural actions – either one could lead to important advantages, such as prolonging play (a correlation found for the studied orangutans). These findings infer an evolutionary continuity of exact facial replications across great apes (and humans) and a complexity for pre-human laugh communication. Laugh faces of ancestral apes must have already been complex in form and function 10–16 million years ago, and later within the hominin lineage become even more effective tools of everyday social communication.

**Supplementary Information:**

The online version contains supplementary material available at 10.1038/s41598-026-43992-w.

## Introduction

Facial replications, where individuals match the facial expressions of others, play a central role in everyday social interactions of humans. Facial replications can be exact, i.e., when the same facial variant type is matched, involving the fine-tuned activation of specific muscles across the social partners^1^. Such a higher degree of matching is likely to correlate with an improved understanding of others, perhaps hereby helping individuals to be more emotionally in tune with others^2–4^ and/or to predict more accurately their behavioural actions^5,6^. Thus, facial replications, especially when carried out in exact ways, may lead to advantages that go beyond those of spontaneously produced facial expressions, such as in promoting social interactions and bonding^7,8^.

A classic example can be found in human smiles. Although various facial features appear to have an emotional impact on the observer (e.g., teeth exposure:^9^, by far the most extensively studied facial features are those that distinguish Duchenne from non-Duchenne smiles. The Duchenne smile is distinct in *orbicularis oculi* muscle activations (causing wrinkles at the eye’s outer corners), leading to it being perceived as the more genuine facial variant of happiness than its counterpart^10,11^. In a study by Krumhuber and colleagues^12^, Duchenne smiles were found to induce more *orbicularis oculi* activations in observers than did non-Duchenne smiles^13^. Such exact replications of Duchenne smiles could help to identify genuine positive emotions of others but also lead to individuals being positively affected^5,14,15^.

In nonhuman primates (from here on ‘primates’), facial replication were studied mainly during social play, contexts that provide a platform for practicing social, cognitive and physical skills within a predominantly benign setting^16,17^, although other contexts were also explored^18^. At this stage in research, the phenomenon of facial replications has been identified across primates and other mammals, especially for rapid facial mimicry (primates: e.g.,^19–23^; other mammals: e.g.,^24–28^). Rapid facial mimicry is measured within one second and has been linked to automaticity in both humans and nonhumans (for an overview, see^16,29^), although there may not always be a response^19,30^. Additionally, facial replications may involve responses that take place after one second, which in humans reflect less automatic processes, including voluntary ones^30^. For nonhuman primates, facial replications were also found to occur after one second and have been referred to as ‘delayed’^31–35^.

Notably less is, however, known about the fine-tuning of facial replications and whether the exact facial replications found in humans may have deep roots in biology. Interestingly, Taylor and colleagues^24^ examined two facial variant types (open-mouth faces with and without upper incisors exposed) in rehabilitant, peer-group living sun bears during play and found that these bears mimicked their playmates in an exact way, i.e., producing the same variant type following the facial variant of the other within one second^24^, a finetuning that may be surprising as these bears tend to be solitary in the wild^36^. Similarly, Bresciani and colleagues^37^ and later Cordoni and colleagues^38^ provided evidence for rapid mimicking of facial variants in zoo gorillas, who lived in small well-defined family groups, comparable in size to the wild^39^. Like for the bears, the examined variants of the gorillas differed in their exposure of upper teeth (present or absent).

In this study, we tested for exact facial replications from an evolutionary perspective by focusing on laugh faces across great apes. Based on the Principle of Maximum Parsimony^40^, a principle that leans on the least number of assumptions for phylogenetic reconstructions where evolutionary scenarios are less likely to destroy and rebuild behaviours, this open-mouth expression of great apes is likely to be a homologue of positive human laugh faces^20,41,42^. Laugh faces of great apes are based on six core activations also found in laughing humans^20,41^: Chimpanzees open their mouths while dropping or stretching their jaws, pulling the lower lip down to expose the lower teeth, and often they also pull the lip corners back and upwards, and raise their upper lips to reveal their upper teeth^20^. Thus, we have here a nonhuman facial expression of play with the same facial muscle activations as positive human laugh faces, that accompanies laugh vocalizations^43^, as in human laugh faces (open-mouth smiles).

In the literature, laugh faces have also been referred to as play faces^44,45^, open-mouth faces^19,46^, and relaxed-open mouth displays (if only the lower teeth are shown^42^. Research on laugh faces has historically focused on two morphologically distinct variants^16^: 1) with upper teeth exposed (comparable with the full play face^35^; 2) without upper teeth exposed (comparable with the relaxed-open mouth display^42^. Their potential signalling of ‘this is play’ or ‘this is just play’ may serve playmates differently, e.g., to coordinate play actions and to avoid escalations and getting hurt^16^; laugh faces with upper teeth exposure seem to predominantly occur during rough/risky play in great apes^22,37,38,45,47^. Laugh faces can also be used differently for different playmate constellations, such as to indicate submissiveness or to reassure the social partner^48–50^. It is also important to consider that facial expressions may sometimes be communicative and other times be more expressing emotions^51,52^.

Previous findings have frequently shown a close relationship between the production of these facial expressions and prolonged play (e.g.,^23,31,33,38,53,54^), where the latter for instance promote an set of cognitive, social, emotional, and motoric skills and perhaps even strengthens their social bonds (see^16,17^). Such potential impact of matching is likely stronger if the replication is more fine-tuned (see^16^). However, given that correlations should not be seen as purely one-directional, we need to consider that prolonged play could also be driven by other factors (e.g., associated with valence, arousal and their physiological bases), which then may lead to replications. As part of this, play bouts, and subsequently longer play bouts, should provide a platform for emotion regulation to become more effective as well as more opportunities for individuals to identify the emotion states of others (see^5,55^), which could lead to an increase in replications. Furthermore, expressions that occur during play have often been linked to positive affect (see^16^), although the negative arousal system may also be involved (see^5^).

In the present work, we tested our hypothesis that exact facial replication is present across great apes, i.e., orangutans and chimpanzees. Orangutans are phylogenetically most distant from humans, and chimpanzees (together with bonobos) are closest. While orangutans have solitary tendencies in the wild^56^, chimpanzees live in large complex social groups^57^. Specifically, we studied a total of 96 great apes from four orangutan groups and four chimpanzee groups using a two-step approach. The first step of this study was to test for facial replications in the subjects by using a previously developed method that controls for play actions and intensities^19^. The second step was to test if these potential replications were exact for both orangutans and chimpanzees. We tested for replications within three seconds as for multimodal laughter (laugh vocalizations plus laugh faces) in the studied chimpanzees, the event peak was found to between two and three seconds^31^. Thus, our response time focus covered both rapid facial mimicry and delayed facial replications^19,31–35^.

Evidence of exact facial replications of both examined taxa would be consistent with the Complexity and Continuity Hypothesis of Laughter and Smiles^41^, which states that laugh faces (and laugh vocalizations) must have been already complex in both form and function in ancestral apes, before they became more effective as pervasive tools in everyday social communication of humans. Furthermore, to explore potential functions of exact replications in this study, we examined laugh face variants in relation to play duration and play intensity. Specifically, we tested if exact facial replications correlated with play duration and whether laugh face variants are associated more with rough versus gentle play.

## Methods

### Subjects and study sites

In this study, we examined 39 orangutans (*Pongo pygmaeus*) living in four separate groups and 57 chimpanzees (*Pan troglodytes*) living in four separate groups. They included immature as well as mature males and females for both the orangutans and the chimpanzees. For a general overview of the number of individuals included in this study and their age and sex classes, see Table 1. For exact replications, altogether 30 orangutans and 39 chimpanzees were tested (for details, see Data Analysis, below). For orangutans, the sample included 7 adolescents/adults (2 females and 5 males) as well as 23 immature subjects (infants and juveniles). For chimpanzees, the sample included 16 adolescents/adults (9 females and 7 males) as well as 23 immature subjects (infants and juveniles). For both taxa, infants were 4 years or below, juveniles were older than 4 years and up to 8 years, adolescents were older than 8 years and up to 12 years and adults were older than 12 years. A detailed overview of the sex class and age of these subjects of each enclosure is available in Supplementary material, Table S1.

**Table 1:** Overview of age and sex classes of the orangutan and chimpanzee subjects and their playmates, respectively. Infants were 4 years or younger, juveniles were > 4 years up to 8 years, adolescents were > 8 years up to 12 years and adults were > 12 years. For the playmates, the number of females is in brackets for adolescents and adults.

	Subjects		Playmates	
			Infants/juveniles	Adolescents/Adults
Orangutans	Infants/juveniles	23	18	3 (0)
	Adult/adolescent females	2	0	3 (1)
	Adult/adolescent males	5	4	3 (1)
Chimpanzees	Infants/juveniles	23	19	9 (4)
	Adult/adolescent females	9	8	3 (2)
	Adult/adolescent males	7	7	3 (2)

The orangutans were stationed at the Sepilok Orangutan Rehabilitation Centre (SORC) nearby Kabili-Sepilok Forest Reserve, Malaysia. These subjects were all rehabilitant orangutans, who arrived as orphans in the rehabilitation centre. The younger orangutans at the nursery stayed indoors at night and they represented two additional groups. These two nursery groups ranged from 1 to 5 years and from 1 to 7 years, respectively. The older orangutans lived in two groups outdoors during the day as part of their rehabilitation training programme. These two outdoor groups ranged from 5 to 13 years and from 9 to 15 years, respectively. The older orangutans were free-ranging and were previously released into the forest by SORC. SORC provided them with food on feeding platforms in the forest two times a day (at around 10am and 3pm), which allowed us to regularly video-record them; nonetheless, the orangutans also occasionally foraged in the forest. The individuals from the nursery at SORC did not interact with the older individuals during the data collection period.

The chimpanzees lived in large outdoor enclosures ranging from 47 to 190 acres outdoor enclosures containing grasslands and forests in the miombo woodland at the Chimfunshi Wildlife Orphanage (CWO), Zambia. We studied four multimale-multifemale semi-wild groups in this environment that enables some fission-fusion dynamics. Each group was composed of a mixture of wild-born chimpanzees and chimpanzees born at CWO (see^31^), with wild-born chimpanzees coming from various sub-species and geographical backgrounds. Similar to the orangutans, the chimpanzees regularly came during feeding times to the feeding areas of their enclosures, where we were able to regularly video-record them. The chimpanzees of this study were fed twice a day (at around 11am and 2pm). The enclosures also contained naturally developed fruit groves (see^58^).

### Video recording collection

The subjects were video-recorded by M.D.-R. and H.C. during spontaneous social play with conspecifics, from August to October 2005 and from July to September 2015 for the orangutans, and from June to August 2007 for the chimpanzees. The chimpanzee and the first orangutan recordings were obtained opportunistically, with the recording starting when play was expected to take place, for instance when two individuals got physically close. The second orangutan data set collected consisted of subjects (different subjects than in 2005) recorded twice a day for 3 min of focal videos. The order of the focal animal sampling was randomized prior to the recording. Only recordings allowing clear visibility of the faces of the individuals were included for analysis. All recordings were obtained with either HD or full HD screen resolutions (720p and 1080p, respectively) and 25 frames per second were continuously used. For this study, we used 209 play bouts for orangutans and 466 bouts for chimpanzees. For both orangutans and chimpanzees, all of the recordings were conducted near their feeding areas.

### Video coding

We identified dyadic play bouts based on bodily play actions displayed by two conspecifics that did not appear to serve any other immediate function. These play actions were systematically coded for play intensity, consisting of gentle play (3 play action types: slow non-tactile play, slow grappling and tickling) and rough play (5 play action types: fast grappling, gnawing, wrestling, hitting and jumping)^19,44^. The ethogram used for the coding is shown in Supplementary material, Table S2. The play bouts started when one individual produced a play action following the play action of another individual, and ended when one individual stopped showing play actions for at least 20  seconds or when a third individual intervened^19,44^. The 20-second threshold was chosen to have a systematic approach to capture dependent play behaviours, although it is possible that longer play intervals are still part of a continuous play session. Only play bouts that lasted more than 20 s were selected for analysis to ensure both playmates were engaged in play^19,20^.

Furthermore, we coded for the presence of open-mouth faces (OMFs), laugh vocalizations and biting (Table S2; also see^19,31,20^. An OMF was identified as a facial expression with a wide parting of the lips, coded as a single event if they had no closed-mouth gaps of 0.5 s or longer^20^. These facial expressions have also been referred to as laugh faces^20,48^ and play faces (see^14,59^). Laugh vocalizations represented a succession of call elements during play with an interval between call elements of no more than 1 s^43^. Biting was coded when the mouth opened and closed in form of snapping, chewing or holding with the jaw. The video coding of the play actions as well as the presence of open-mouth faces (OMFs), laugh vocalizations and biting were previously completed for the first orangutan video collection and for the chimpanzees (Table S2; also see^19,20,31^. We used the same coding scheme for the second orangutan video collection, so that the same coding approach was applied for all the videos included in the current study.

**Fig. 1:**
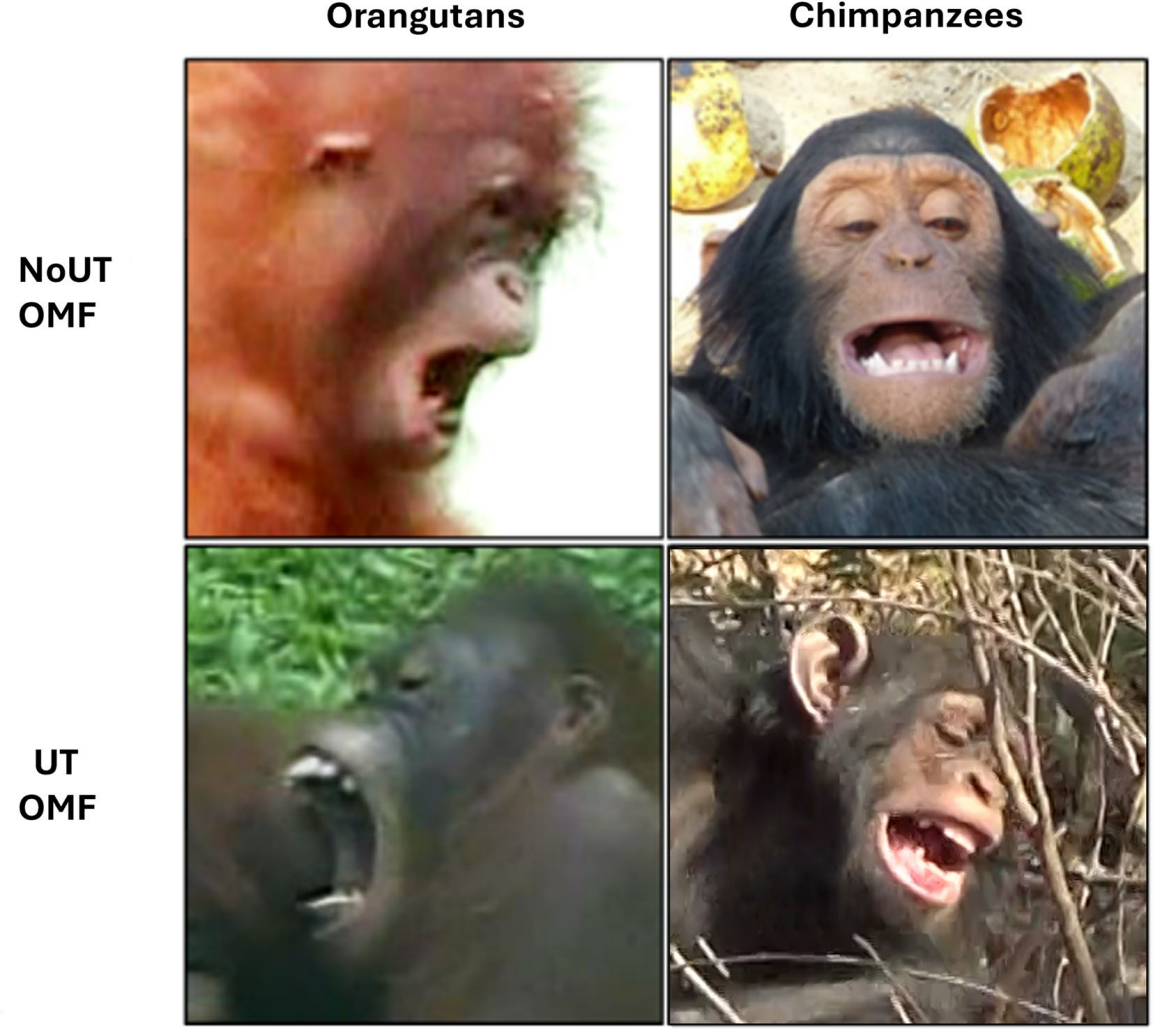
Two variants of OMFs (NoUT OMF and UT OMF) in orangutans (left panels) and chimpanzees (right panels).

Additionally, we identified the OMF variants by determining if the OMF showed no-upper-teeth exposure (i.e., NoUT OMF) (*n* = 698 in orangutans and *n* = 848 in chimpanzees) or upper-teeth exposure (i.e., UT OMF) (*n* = 585 in orangutans and *n* = 242 in chimpanzees) (see Fig. 1 for a distinction between OMF variants); UT OMF is comparable to full play faces (e.g.,^35^), NoUT is comparable to relaxed-open mouth displays (e.g.,^42^). We used the terms NoUT and UT OMF due to their link to morphology. For all play bouts, we also coded if the dyad showed facing, i.e., when both individuals positioned their faces at an angle of 45 degrees or less directed towards each other (for facial directedness, see^60^; in cases where only one individual was positioned this way, facing was considered absent (Table S2).

Altogether five main coders contributed to this study, focusing on a behavioural category (e.g., play actions) at a time. Importantly, every playing great ape was coded separately (except for facing) and the OMF coders were not aware of the aim of this study, reducing the potential of any bias. Video coding was conducted with Interact 8 (Mangold, Arnstorf, Germany), where we assessed every 4 frames (0.16 s) if behavioural changes occurred (25 frames per second). For the inter-coder reliability testing, we calculated Cohen’s kappa based on 15% of 2373 OMFs, with scores ranging from good to excellent. We obtained k = 0.79 and 0.83 for the OMF variants as well as k = 0.85 and 0.88 for facing, regarding the orangutans and chimpanzees, respectively. In addition, reliability was excellent for play actions (k = 0.83 and 0.89) and laugh vocalizations (k = 0.92 and 0.95), calculated based on 15% of 675 play bouts (*n* = 466 for chimpanzees and *n* = 209 for orangutans, respectively). Two coders carried out the coding for the reliability test with three other coders.

### Data analyses

In order to test for exact facial replications, the first step was to test for overall facial replications (i.e., to determine if the apes of this study showed open-mouth responses in general) and the second step was then to explicitly test for exact facial replications (i.e., to test the hypothesis). One-tailed tests were used throughout these two steps. The latency of potential OMF replications was also measured (from the onset of the playmate OMF to the onset of the subject OMF). For an illustration of facial exchanges, see Supplementary material, Fig. S1. Furthermore, we explored OMFs in relation to play duration as well as play intensity.

#### Testing for exact facial replications (hypothesis)

1) To test for overall facial replications, we extracted Display scenes, i.e., scenes where one of the playing individuals was showing an OMF and both individuals were facing each other for at least 3 s, following the onset of this OMF. With the 3-second time frame, we therefore covered both the time frame used to study rapid facial mimicry (e.g.,^19–23^) as well as more delayed responses^31–35^.

The individual showing the OMF at the scene onset was identified as the ‘playmate’, and the other individual was the ‘subject’. Each Display scene was paired with a No-Display scene, i.e., scenes where the playmate was not producing an OMF at the scene onset. Prerequisites for the No-Display scene were that both scenes showed the same subject and playmate facing each other for at least 3 s, the same play action types (and consequently the same play intensity). For example, if the Display scene showed subject *A* with play action *x* and playmate *B* with play action *y*, then the No-Display scene needed to also show *A* with *x* and *B* with *y*. If laugh vocalizations were absent in the Display scene, then they also needed to be absent in the No-Display scene.

To find a Display scene, we started our search from the beginning of a play bout. Once the Display scene was found, we started our search for the No-Display scene at least 5 s apart (to ensure it did not overlap with potential replications) and from there onwards throughout the rest of the play bout. If the latter scene could not be found within the same play bout, the search continued within another play bout. In such rare cases, the potential difference in social context for instance could have introduced a bias. The combination of Display and No-Display scenes allowed us to control for potential confounding effects, including play intensity or biting, when assessing the presence of exact facial replication. Indeed, biting actions show a wide opening of the mouth similar to OMFs and both may occur in sequence, but it is also possible that biting triggers an opening of the mouth in the other playmate^19^. As a consequence, scenes showing biting were excluded from the analysis. Furthermore, the combined scenes (Display and No-Display) were measured for the same duration.

The combination of Display and No-Display scenes led to four potential scenarios: the subject shows an OMF only in the Display scene (i.e., congruent behaviours), the subject shows an OMF only in the No-Display scene (i.e., non-congruent behaviours), the subject shows an OMF in both scenes, and the subject shows an OMF in none of the scenes^19,24^ (Table 2). To test for facial replications, the numbers of subjects showing congruent behaviours and non-congruent behaviours were compared. For this step, we used McNemar’s tests (nonparametric), which allowed us to test the paired binary data. To avoid pseudo-replications, it was reported for this test if a subject was ever observed to show a congruent behaviour and/or non-congruent behaviour. A total of 66 subjects were included in this analysis (*N* = 29 orangutans and 37 chimpanzees).

**Table 2:** The four scenarios of Display and No-Display scenes and their number of subjects for both orangutans and chimpanzees: The subject shows an OMF only in the Display scene (i.e., congruent behaviours), the subject shows an OMF only in the No-Display scene (i.e., non-congruent behaviours), the subject shows an OMF in both scenes, and the subject shows an OMF in none of the scenes. The number of events are shown in brackets.

4 scenarios	Playmate face	Subject face	Orangutans: Number of subjects (events)	Chimpanzees: Number of subjects (events)
Congruent behaviours	Display scene: OMF	OMF	29 (113)	37 (274)
	No-Display scene: No OMF	No OMF		
Non-congruent behaviours	Display scene: OMF	No OMF	0 (0)	0 (0)
	No-Display scene: No OMF	OMF		
Always producing OMFs	Display scene: OMF	OMF	16 (30)	14 (29)
	No-Display scene: No OMF	OMF		
Never producing OMFs	Display scene: OMF	No OMF	20 (66)	30 (156)
	No-Display scene: No OMF	No OMF		

2) We then explicitly tested for exact facial replications with Wilcoxon signed-ranks tests (hypothesis). We compared the number of times each subject matched the OMF variant of their playmate to the number of times each subject produced the non-exact variant, by calculating OMF rates. There were two possibilities in which the subject could show an exact variant and two possibilities in which the subjects could show a non-exact variant:

- Playmate UT OMF → subject UT OMF (subject shows an exact variant)
- Playmate NoUT OMF → subject NoUT OMF (subject shows an exact variant)
- Playmate UT OMF → subject NoUT OMF (subject shows a non-exact variant)
- Playmate NoUT OMF → subject UT OMF (subject shows a non-exact variant)

A total of 69 subjects were included (*N* = 30 orangutans and 39 chimpanzees) when testing for exact facial replications. The sample size for each analysis depended on the playmates’ OMF variants.

#### Play duration and play intensity

We examined whether play bout duration was associated with the likelihood that the subjects produced the same OMF variant as the preceding OMF of their playmate. The occurrence was calculated based on the subject showing the same variant versus the different variant in comparison to the playmate’s variant. All subjects who included in the exact replication analyses were tested here as well (*N* = 30 orangutans and 39 chimpanzees). Then, we examined the relationship between play intensity and any matching of OMFs in the subjects, i.e., the mean occurrence rate of producing the same OMF variant and producing the different OMF variant following the playmates OMF. The sample size was reduced in different ways for each test due to the separate analyses for gentle play and rough. Finally, we examined the mean occurrence rate for each OMF variant per minute of play by comparing these rates during rough play versus during gentle play. For this analysis, we included all individuals of this study, including playmates and including playing individuals who produced no OMFs, in order to obtain occurrence rates that best represent the studied apes’ natural behaviours (*N* = 39 orangutans and 57 chimpanzees). To measure play duration and play intensity, we used Spearman’s rank-order correlation tests and Wilcoxon signed-ranks tests, respectively.

When Wilcoxon signed-ranks tests were applied in this study, the pairing of related samples led to the removal of missing data, which differed across the variables being compared. Hommel-Hochberg corrections^61^ were applied to adjust α for multiple comparisons. The statistical analyses were carried out using SPSS Statistics 26 (IBM, Chicago, IL, USA).

## Results

### Testing for facial replications

First, we tested for overall facial replications. Significantly more orangutans showed congruent OMFs than orangutans who showed non-congruent OMFs (one-tailed McNemar’s test: χ^2^ = 14.06, N*total* = 29, p ˂ 0.001; Table 2). Similarly, significantly more chimpanzees showed congruent OMF behaviours than non-congruent OMF behaviours (χ^2^ = 12.07, N*total* = 37, *p* < 0.001; Table 2).

**Fig. 2:**
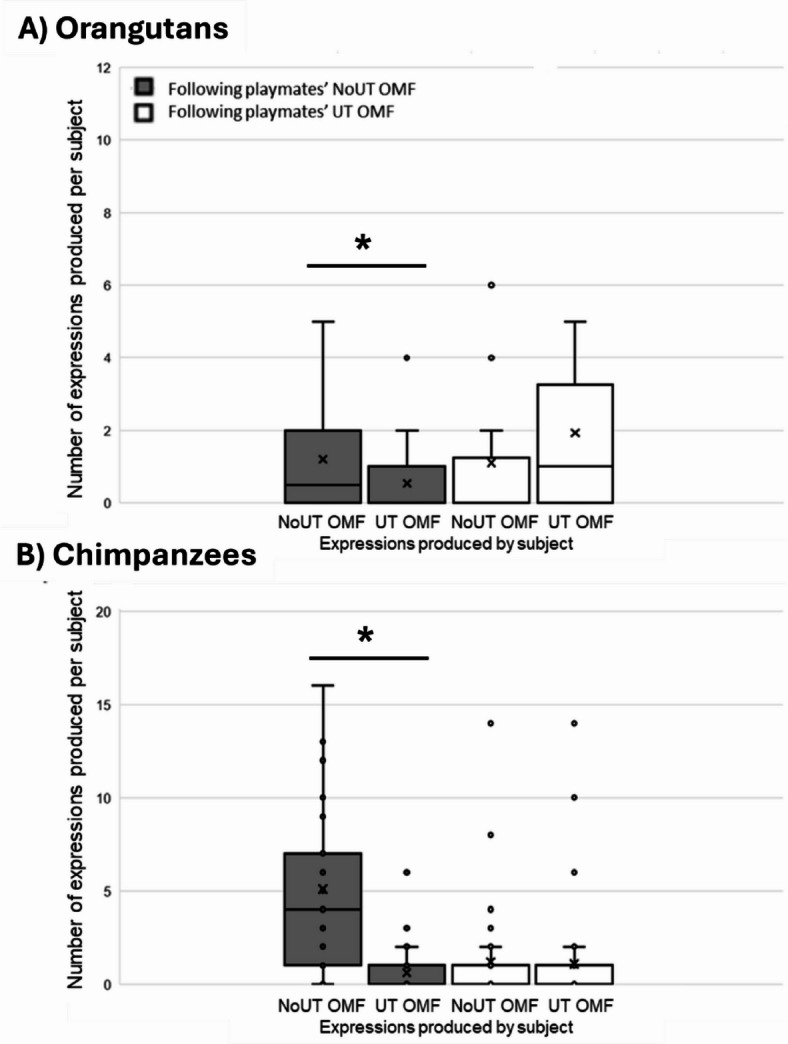
The total number of great ape NoUT OMF and UT OMF expressions following the playmate’s NoUT OMF expressions and UT OMF expressions per subject, for (**A**) the orangutans and (**B**) the chimpanzees. The box plots depict medians (horizontal lines within the box), means (crosses), upper and lower quartiles, and minimum and maximum range values. I.

Next, we tested for exact facial replications (hypothesis). Following playmates’ NoUT OMF expressions, the orangutan subjects produced significantly higher mean rates of NoUT OMF expressions than mean rates of UT OMF expressions (one-tailed Wilcoxon signed-ranks, Z = -1.798, N*test* = 18, *p* = 0.036), but they showed no differences in rates of OMF variant following the playmates’ UT OMF expressions (Z = -0.882, N*test* = 24, *p* = 0.189). Similarly, following playmates’ NoUT OMF expressions, the chimpanzee subjects produced significantly higher rates of NoUT OMF expressions than UT OMF expressions (one-tailed Wilcoxon signed-ranks, Z = -4.593, N*test* = 38, *p* < 0.001), but they also showed no such differences following the playmates’ UT OMF expressions (Z = -0.762, N*test* = 20, *p* = 0.223). For an overview of the total numbers of OMF variants of the subjects following OMF variants of the playmates, see Fig. 2.

Furthermore, the orangutan mean latency was calculated to be 1.1 ± 0.8 SD seconds (1.2 ± 0.8 for NoUT OMF and 0.9 ± 0.6 for UT OMF), with 54.8% of the subject OMFs occurring within 1 s following the playmate OMF. The chimpanzee mean latency was 1.1 (± 0.7 SD) seconds (1.1 ± 0.9 SD for NoUT OMF and 1.1 ± 0.8 SD for UT OMF), with 64.5% of the subject OMFs occurring within 1 s following the playmate OMF.

### Play duration

**Fig. 3:**
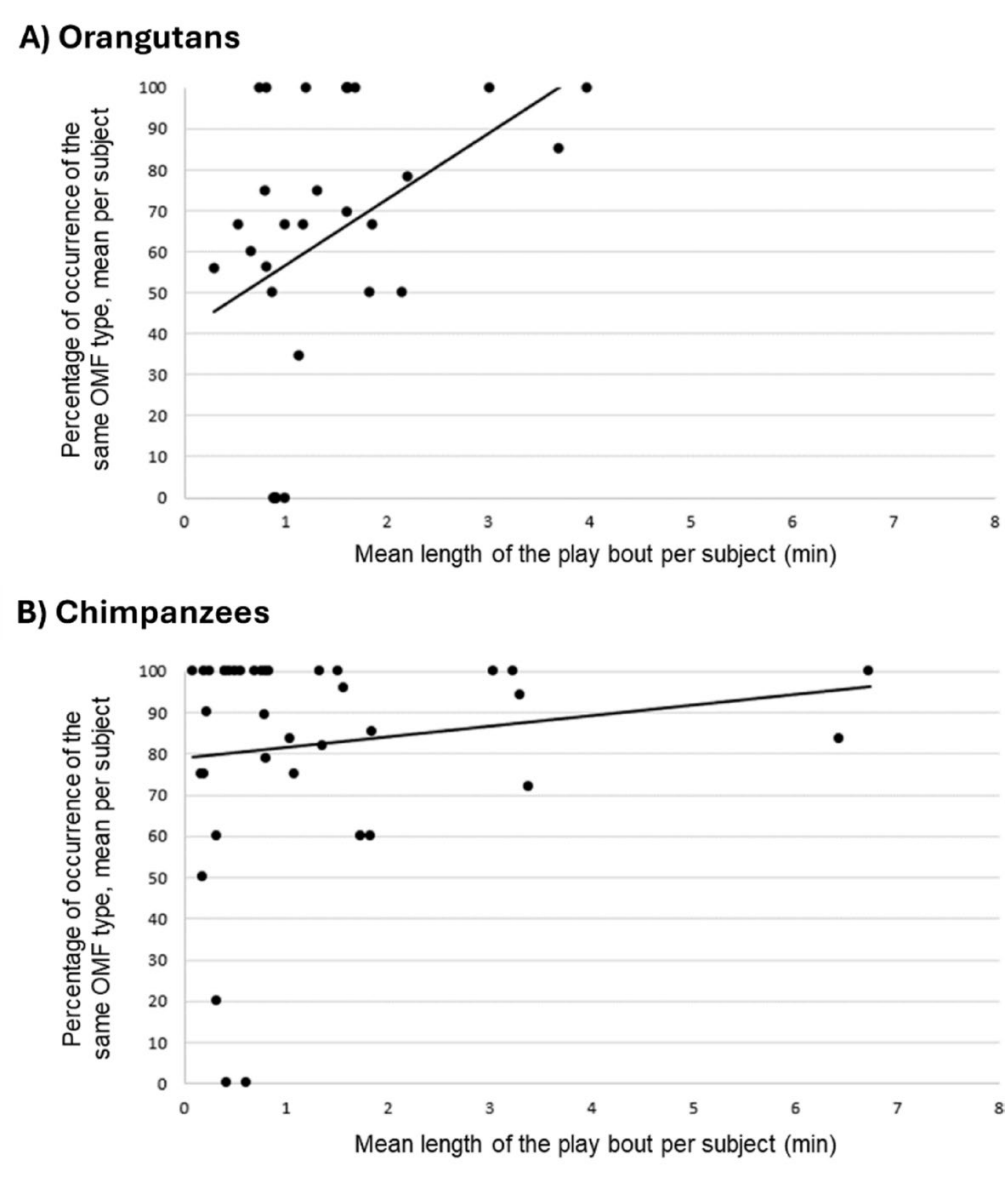
Percent occurrence of the same OMF variant in the subjects following the playmates’ OMF variant, as a function of play bout duration (**A**) For orangutans, 30 subjects were included. (**B**) For chimpanzees, 39 subjects were included.

We then examined the relationship between play bout duration and the mean percent occurrence of the subject matching the preceding playmate OMF variant. The results showed a borderline significant positive relationship for the orangutans, where the subjects who played longer also showed a higher percent occurrence of producing same OMF variant (two-tailed Spearman’s rank-order correlation tests; r_s_ = 0.36; N*total* = 30; *p* = 0.050; Fig. 3A). No such relation was found for the chimpanzees (r_s_ = 0.041; N***total*** = 39; *p* = 0.810; Fig. 3B). The orangutan play bouts lasted a mean of 1.3 (± 1.5 SD) minutes, while the chimpanzee play bouts lasted a mean of 1.4 (± 1.0 SD) minutes.

### Play intensity

We examined both rough and gentle play with regard to the mean occurrence rate of matching, i.e., the subjects producing the same (versus different) OMF variants as the playmates. During rough play, the chimpanzee subjects produced significantly more often the same OMF variant as the playmates than the different OMF variant (two-tailed Wilcoxon signed-ranks, Z = -3.132, N*test* = 28, *p* = 0.002) and they significantly more often matched the NoUT OMF than the UT OMF of the playmates (Z = -2.285, N*test* = 15, *p* = 0.022). During gentle play, the chimpanzee subjects also matched more often the OMF variant of their playmate than not (Z = -4.518, N*test* = 32 *p* < 0.001) and significantly more often matched the NoUT OMF than the UT OMF of the playmates during gentle play (Z = -2.294, N*test* = 10, *p* = 0.022). In contrast, during rough play, the orangutan subjects showed a tendency of producing more often the same OMF variant as the playmates than the different OMF variant (two-tailed Wilcoxon signed-ranks, Z = -1.941, N*test* = 29, *p* = 0.052), but they did not match more often one variant over the other (Z = -1.199, N*test* = 27, *p* = 0.842). During gentle play, the orangutan subjects showed no indication of matching the OMF variant of their playmates (Z = -0.447, N*test* = 5, *p* = 0.655).

Finally, we examined the mean occurrence rate for each OMF variant during rough and gentle play. The chimpanzees (N*total* = 57) produced significantly more often UT OMFs per minute of rough play than per minute of gentle play (two-tailed Wilcoxon signed-ranks, Z = -3.075, *p* = 0.002), but they showed no such difference for NoUT OMF rates (Z = -1.558, *p* = 0.120). The orangutans (N*total* = 39) showed no such difference for neither UT OMF rates (Z = -1.312, *p* = 0.193) nor NoUT OMF rates (Z = -0.487, *p* = 0.633).

## Discussion

The present study tested for exact facial replications in great apes by assessing orangutans and chimpanzees during social play. Based on the Principle of Maximum Parsimony^40^, where evolutionary scenarios are less likely to destroy and rebuild behaviours, these facial expressions are likely to represent precursors of human laugh faces and smiles^20^; for a discussion on the evolutionary reconstruction of smiles, see^41^.

Our first step was to test if the orangutans and chimpanzees of our study showed facial replications in general, independent of whether they were exact or not. Our results showed that significantly more orangutans and chimpanzees produced congruent behaviours than the respective great apes produced non-congruent behaviours within the three seconds following the playmates’ laugh faces. We argue that play intensity is less likely to represent a confound in these results as we compared scenes of the same play actions when testing for such matching, consequently controlling for play intensity. Our findings, therefore, revealed a pattern of rapid and delayed replications in the orangutans and chimpanzees, where the laugh faces of playmates triggered laugh faces in the observing subjects. These findings align with previous ones on primates and other mammals (e.g.,^19–28,31–35^).

For the second step, we tested the exact facial replication hypothesis by examining if the great apes replicated the specific laugh face variant displayed by their playmates. We found that after the playmates displayed laugh faces without upper teeth exposure, both orangutan and chimpanzee subjects produced more often the same variant type (i.e., NoUT OMF) than the other one (i.e., UT OMF). As such, the current study provides strong empirical evidence for exact facial replications in orangutans and chimpanzees, as found in humans^1,12^. In humans, the benefit of replicating Duchenne smiles in an exact way may be to identify genuine positive emotions, but it could also be that it leads to individuals being positively affected, or both^5,12–15^.

Since orangutans and chimpanzees occupy opposite ends of the great ape phylogenetic clade, the most parsimonious explanation for our findings is that exact facial replication is an evolutionary continuous phenomenon. This claim is further strengthened by comparable outcomes on gorillas^37,38^ as well as findings on humans, indicating that the degree of laugh face teeth exposure may affect emotional information processed by observers^9^– although these facial features require further attention in human research. As such, we argue that this ability of fine-tuning as part of a laugh face exchange among social partners must have played an important role already when the last common ancestor of great apes and humans existed, 10–16 million years ago. It reveals a complexity that most likely benefitted ancestral and extant great apes, supporting the Complexity and Continuity Hypothesis of Laughter and Smiles^41^, which states that the precursors of human laugh faces and laugh vocalizations must have been complex not only in form but also in social function.

On a related matter, for the orangutans of our study, facial replications became more exact with prolonged play bouts. On one hand, this relationship could be explained by the possibility that such replications may help strengthen their social bonds and promote a set of cognitive, social, emotional, and motoric skills^16,17^. This possible explanation resembles previous ones where similar findings on primates were obtained, albeit for non-exact replications (e.g.,^19–23,31–33,53,54^). On the other hand, it is also possible that play itself or factors linked to playing longer (e.g., increased arousal and physiological changes) may induce these replications. For example, longer play bouts may enhance the effectiveness of emotion processing and offer more opportunities to identify emotion states of social partners^5,55^, which could then increase the fine-tuning in facial replications. While this topic deserves further research attention, it is likely that being in sync with conspecifics may benefit some primates particularly – such as individuals or taxa that do not live in family groups (e.g., peer-grouped rehabilitant orangutans; see[^62^]).

Furthermore, the chimpanzees of this study matched the laugh faces without upper teeth exposure more often than the other variant type, independent of whether they played roughly or gently. This preference explains at least partly why the chimpanzees (and orangutans) showed exactness in facial replications when the upper teeth were not exposed, whereas the matching of exposed teeth was infrequent. As the upper teeth exposure is linked with rough play in our study’s chimpanzees (consistent with studies on other great apes^16,22,37,45,47^, these facial features may signal risky play that might be disadvantageous to match – at least when playing with older individuals or males^48^ and when the groups are large and also include non-kin members (e.g., the chimpanzee groups of this study; see^31^.

Interestingly, zoo gorillas were found to rapidly mimic (≤ 1 s) such upper teeth exposure in play^37,38^, perhaps because they lived in smaller family groups. Rearing conditions may also affect great ape play behaviours^63–65^. On a further note, great ape mothers may intervene when play becomes too rough^44,63^, possibly influencing what the youngsters come to associate with an upper teeth exposure. Indeed, mother-infant interactions seem to be relevant for the development of mimicking behaviours in non-human primates^32,34,52,66^ and humans^67^. Moreover, differences found within our study might also be partly explained by taxon-specificity. For example, primate species with larger typical group sizes tend to have greater facial mobility, i.e., the variety of facial movements a species can produce^68^. Wild chimpanzees typically live in large multi-male multi-female groups^57^, whereas wild orangutans form small social units^56^, albeit smaller than the family groups of wild gorillas^39^. Furthermore, in sun bears (mammals with solitary tendencies), evidence for exact replications was also found for both variant types that differed in upper teeth (incisor) exposure^24^.

It is important to note that the differences between our study and the gorilla studies^37,38^ cannot be explained with our different response time focus as our study captured both rapid (≤ 1 s) and delayed responses (> 1 to 3 s) at similar rates and revealed no bias towards any particular laugh face variant type. Further methodological explanations are also unlikely since we obtained evidence for exact replications regarding laugh faces without upper teeth exposure.

All in all, the present work revealed that laugh faces are used in subtle ways to communicate as part of an exact facial exchange among social partners in the two phylogenetically most distanced taxa among the extant great apes (orangutans and chimpanzees). These findings suggest an evolutionary continuity of behaviour not only in form but also in function, a pattern that is further supported by previous gorilla findings^37,38^. With humans showing comparable abilities^1,9,21,13^, we argue that it must have been advantageous to replicate laugh faces in exact ways already when the last common ancestor of great apes and humans existed. Especially the less risky variant type, i.e. with covered upper teeth, may be important to replicate in exact ways, possibly to be in tune with others (see^4^) and/or to predict their behaviours^5^, which could promote social cohesion, coordination as well as different sets in skill development^6,8,16,17^. It is possible that the effect is similar to how smile variants in humans affect perceived happiness^1,9,12^, but this requires further investigation.

Our findings reveal a complexity in laugh communication in both form and function that is consistent with the Complexity and Continuity Hypothesis of Laughter and Smiles^41^. As such, we argue that laugh faces of ancestral ape 10–16 million years ago must have been complex in form and function, around the time when laugh vocalizations already existed^20,43,69^. From this point onward, these expressions may have become even more effective within the hominin lineage, where they increasingly served as pervasive tools of everyday social communication.

## Supplementary Information

Below is the link to the electronic supplementary material.

Supplementary Material 1

## Data Availability

The datasets used during the current study available from the corresponding author on reasonable request.
