# Supplementary material for: Towards the complexity of laugh communication in great apes: exact facial replications in laugh faces of orangutans and chimpanzees
Source: Sci Rep. 2026 Mar 14;16:11758. doi: 10.1038/s41598-026-43992-w (PMC13066440; doi:10.1038/s41598-026-43992-w)
Supplement: Supplementary file 1 — Supplementary Material 1 [file 41598_2026_43992_MOESM1_ESM.docx]

# SUPPLEMENTARY MATERIAL

Table S1. Overview of the sex class and age (in years) of the subjects of each enclosure.

| **Orangutans** | **Age** | **Chimpanzees** | **Age** |
| --- | --- | --- | --- |
| **Nursery group, 2005** | **N=11** | **Group 1, 2007** | **N=11** |
| 2 females | 3 | 2 females | 7 |
| 2 females | 4 | 1 female | 11 |
| 2 females | 5 | 1 female | 13 |
| 1 male | 1 | 2 male | 3 |
| 2 males | 3 | 1 male | 6 |
| 2 males | 4 | 1 male | 7 |
| **Nursery group, 2015** | **N=6** | 1 male | 8 |
| 1 male | 1 | 1 male | 14 |
| 1 male | 2 | 1 male | 16 |
| 2 males | 3 | **Group 2, 2007** | **N=17** |
| 1 male | 5 | 1 female | 3 |
| 1 male | 7 | 1 female | 3 |
| **Outdoor group, 2005** | **N=8** | 2 females | 4 |
| 1 male | 5 | 1 female | 5 |
| 2 males | 6 | 2 females | 6 |
| 2 males | 7 | 1 female | 9 |
| 2 males | 9 | 1 female | 10 |
| 1 male | 13 | 1 female | 17 |
| **Outdoor group, 2015** | **N=5** | 1 female | 30 |
| 1 female | 15 | 1 female | 35 |
| 1 female | 19 | 1 male | 1 |
| 1 male | 9 | 1 male | 3 |
| 1 male | 9 | 2 males | 6 |
| 1 male | 10 | 1 male | 7 |
|  |  | **Group 4, 2007** | **N=1** |
|  |  | 1 male | 25 |
|  |  | **Orphanage group, 2007** | **N=10** |
|  |  | 1 female | 2 |
|  |  | 1 female | 5 |
|  |  | 1 female | 7 |
|  |  | 1 female | 8 |
|  |  | 1 female | 9 |
|  |  | 2 males | 5 |
|  |  | 1 male | 6 |
|  |  | 1 male | 10 |
|  |  | 1 male | 30 |

Table S2. Ethogram with the list and definition of behaviours coded for open-mouth faces, laughter and play actions (based on Davila-Ross et al. 2008; Flack et al. 2004).

| **Open-mouth faces** | |
| --- | --- |
| Upper-teeth OMF (UT OMF) | OMF variant with upper teeth exposed |
| No-upper-teeth OMF (NoUT OMF) | OMF variant showing no upper teeth exposure |
| **Laughter** | Succession of call elements during play with an interval between the calls of 1 second or less. |
| **Biting** | Mouth opening and closing in form of snapping, chewing or holding with the jaw. |
| **Facing** | The playmates were facing each other with a maximum angle of 45 degrees of head rotation, and with a free vision of each other’s face (not physically obstructed) at the onset of the response of the recipient |
| **Play actions** | |
| Slow nontactile | Slow movement play of no physical contact, e.g., slapping hands on the ground while sitting opposite the other playmate; a form of gentle play |
| Slow grappling | Holding the other playmate with hands/feet and moving/pulling slowly and relaxed; a form of gentle play |
| Tickling | Gently poking the other playmate with moving fingers/hand; a form of gentle play |
| Fast grappling | Holding the other playmate with hands/feet and moving/pulling quickly and abruptly; a form of rough play |
| Gnawing | Chewing some body part of the other playmate; a form of rough play |
| Wrestling | Rough and tumbling while in close bodily contact with the other playmate; a form of rough play |
| Hitting | Slapping the other playmate; a form of rough play |
| Jumping | Bouncing on/off the other playmate; a form of rough play |


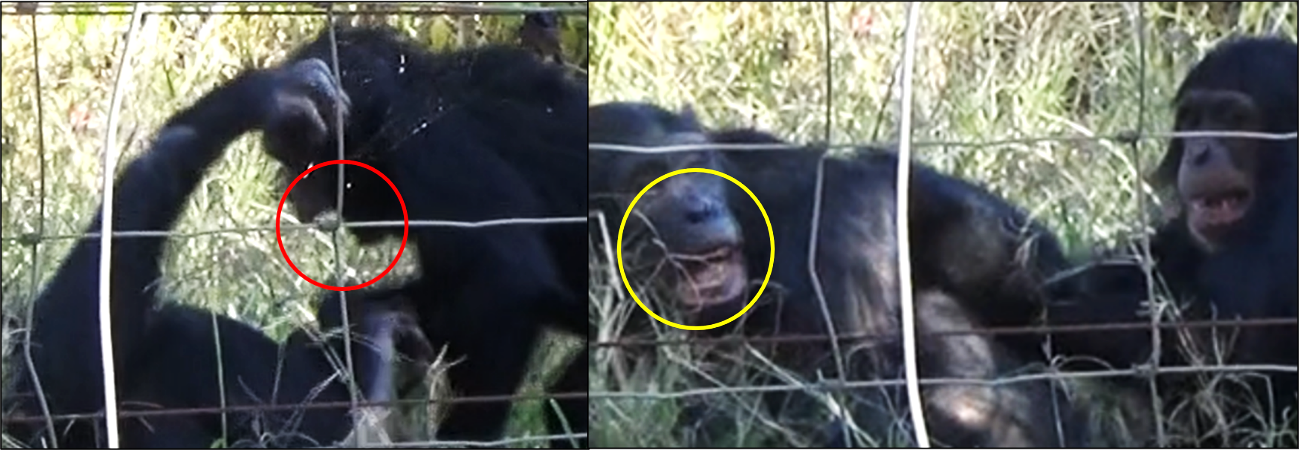


**Figure S1.** Screenshots of facial exchanges in two chimpanzees. The OMF of the playmate is circled in red, and the OMF of the subject is circled in yellow.
